# Higher expression of miR-133b is associated with better efficacy of erlotinib as the second or third line in non-small cell lung cancer patients

**DOI:** 10.1371/journal.pone.0196350

**Published:** 2018-04-24

**Authors:** Alessandra Bisagni, Maria Pagano, Sally Maramotti, Francesca Zanelli, Martina Bonacini, Elena Tagliavini, Luca Braglia, Massimiliano Paci, Andrea Mozzarelli, Stefania Croci

**Affiliations:** 1 Pathology Unit, Azienda Unità Sanitaria Locale - IRCCS di Reggio Emilia, Reggio Emilia, Italy; 2 Oncology Unit, Azienda Unità Sanitaria Locale - IRCCS di Reggio Emilia, Reggio Emilia, Italy; 3 Clinical Immunology, Allergy and Advanced Biotechnologies Unit, Azienda Unità Sanitaria Locale - IRCCS di Reggio Emilia, Reggio Emilia, Italy; 4 Scientific Directorate, Azienda Unità Sanitaria Locale - IRCCS di Reggio Emilia, Reggio Emilia, Italy; 5 Thoracic Surgery Unit, Azienda Unità Sanitaria Locale - IRCCS di Reggio Emilia, Reggio Emilia, Italy; 6 Department of Food and Drug, University of Parma, Parma, Italy; Seoul National University College of Pharmacy, REPUBLIC OF KOREA

## Abstract

Epidermal growth factor receptor (EGFR) tyrosine kinase inhibitors (gefitinib, erlotinib and afatinib) are indicated as first-line therapy in patients with non-small cell lung cancer (NSCLC) whose tumors harbor activating mutations in the EGFR gene. Erlotinib is also used in second and third-line therapy for patients whose tumors have wild type EGFR but to date there are no validated biomarkers useful to identify which patients may benefit from this treatment. The expression level of four miRNAs: miR-133b, -146a, -7 and -21 which target EGFR was investigated by real-time PCR in tumor specimens from NSCLC patients treated with erlotinib administered as the second or third line. We found that miR-133b expression level better discriminated responder from non-responder patients to erlotinib. Higher levels of miR-133b in NSCLCs were associated with longer progression-free survival time of patients. Functional analyses on miR-133b through transfection of a miR-133b mimic in A549 and H1299 NSCLC cell lines indicated that increasing miR-133b expression level led to a decreased cell growth and altered morphology but did not affect sensitivity to erlotinib. The detection of miR-133b expression levels in tumors help in the identification of NSCLC patients with a better prognosis and who are likely to benefit from second and third-line therapy with erlotinib.

## Introduction

Non-Small Cell Lung Cancer (NSCLC) is the second most common cancer and is by far the leading cause of cancer death among both men and women [1].

Studies about the molecular characterization of NSCLC showed an important role of specific genes such as those encoding the ErbB protein family. This family includes four plasma membrane receptors: HER-1 (epidermal growth factor receptor, EGFR or ErbB-1), HER-2/neu (ErbB-2), HER-3 (ErbB-3) and HER-4 (ErbB-4). After ligand binding, the receptors form homodimers or heterodimers, that internalize and autophosphorylate tyrosine residues in their cytoplasmic domain, triggering a cascade that leads to cellular proliferation, invasion, metastasis, and inhibition of apoptosis [2–4].

In particular, NSCLC is one of epithelial cancers generally characterized by high expression levels of EGFR and its ligands, frequently carrying activating mutations in exon 18, 19 and 21 of EGFR. As a consequence, tyrosine kinase inhibitors (TKIs) targeting EGFR (gefitinib, erlotinib and afatinib) have emerged as effective drugs for therapy of NSCLC [5–7]. Harbouring activating mutations in EGFR is one of the indications for the use of EGFR-TKIs as first-line therapy. Indeed, the IPASS phase III randomized trial demonstrated better outcome with first-line EGFR-TKI treatment in patients with EGFR-mutant NSCLC compared with platinum-based chemotherapy [8].

However, up to 15% of patients with wild type EGFR NSCLCs can effectively respond to EGFR-TKIs. The TITAN study compared erlotinib versus chemotherapy (docetaxel or pemetrexed) in patients with disease progression during or immediately after 4 cycles of first-line platinum-based chemotherapy. It was found no significant difference in efficacy between erlotinib and chemotherapy in this poor prognosis patients [9]. Based on this data, erlotinib was approved as an alternative to chemotherapy in second-line treatment, regardless of EGFR mutational status and considering patients’ preferences and specific toxicity risk profiles [10–17]. Erlotinib, in second/third-line setting, has shown a significant improvement in median survival, quality of life, and related symptoms in an unselected population with advanced and metastatic NSCLC. Furthermore, the erlotinib efficacy and clinical benefit were demonstrated in a randomized phase III trial of 731 patients with stage IIIB-IV NSCLC. Chemotherapy non-responder patients were assigned to receive erlotinib 150 mg daily or placebo and treatment with erlotinib resulted in improved survival, progression-free survival and response [13, 18, 19].

The identification at diagnosis of which patients with wild type EGFR are more likely to benefit from EGFR-TKIs is still an unmet clinical need. The utility of the evaluation of EGFR protein levels and gene copy number to predict responders and non-responders is still controversial [20]. Overall, to date there are no reliable and validated biomarkers to select patients with wild type EGFR who have better chances to respond to EGFR-TKIs.

MiRNAs are small, non-coding RNAs able to down-regulate expression of multiple proteins mainly through inhibition of translation and induction of degradation of multiple mRNAs recognized by base pairing [21]. Alterations of miRNA expression have increasingly been associated with pathological changes of cancer cells, indicating miRNAs to be one of the molecules that need to be identified. Moreover, some miRNA are known to regulate EGFR pathway in lung cancer and may affect EGFR-TKIs sensitivity as well as patients’ outcome [22–25]. In addition, several studies have shown that miRNAs can help to sub-classified NSCLC and may also predict prognosis and disease recurrence in NSCLC [26–31].

In the present study we investigated the potential of four miRNAs targeting EGFR to predict response to second and third-line therapy with erlotinib in patients with NSCLC based on their expression level in tumor specimens at diagnosis. Further we focused our attention on miR-133b, the most promising miRNA, exploring the possible role that it might play in the sensitivity to erlotinib in lung cancer cell lines.

## Material & methods

### Patients and sample collection

Patients with lung adenocarcinoma in an advanced stage who received Erlotinb as second- or third-line therapy from January 2009 to December 2014 were included in the study. The tumor tissues were fixed in 10% neutral-buffered formalin and stored as paraffin-embedded (FFPE) at Pathology Unit Arcispedale S. Maria Nuova—IRCCS of Reggio Emilia. All patients had FFPE tumor samples. Exclusion criteria included concomitant primary cancer in other sites, comorbidities that contraindicate erlotinib treatment and incomplete clinical data.

Tumour histologic grade was assessed according with the World Health Organization criteria (2015) [1]. All tissues were stained with haematoxylin and eosin for histologic examination by pathologist. Tumours were staged according with the 7^th^ edition of the TNM staging system of the American Joint Committee on Cancer [32]. Objective tumor response was determined using Response Evaluation Criteria in Solid Tumours (RECIST Version 1.1). The patients with disease stabilization on erlotinib for at least 6 months were considered responders. Overall survival time (OS) was measured from the date of diagnosis to the date of death from any cause. Progression free survival (PFS) was defined as the time from erlotinib treatment start to the first observation of objective disease relapse or progression or death due to any cause.

The study methodologies were conformed to the standards set by the Declaration of Helsinki, and the study was approved by the Arcispedale “S.Maria Nuova—IRCCS” Ethical Committee of Reggio Emilia (protocol #143/2014). Written inform consent was obtained from all patients.

### Cell lines

The two non-small lung cancer cell lines A549 and H1299 were routinely maintained at 37°C with 5% CO_2_ and cultured in RPMI-1640 medium supplemented with 10% fetal bovine serum, 100 U∙ml^-1^ pennicillium, and 100 U∙ml^-1^ streptomycin sulfate (Gibco, Carlsbad, CA, USA). H1299 has wild-type KRAS while A549 has mutant KRAS. Both cell lines have wild type EGFR and are not sensitive to erlotinib treatment.

### RNA isolation and real time PCR

Total RNA was extracted from 5 paraffin-embedded tissues sections of 5 μm thickness using High Pure miRNA Isolation Kit (Roche). Total RNA was extracted from cell lines using mirVana RNA Isolation Kit (Ambion, Life Technologies) according to the manufactures’ instruction. Nucleic acid concentration was determined by measuring the absorbance at 260 nm with NanoDropTM 1000 (Thermo Fisher Scientific) instrument. Ten ng of total RNA were reverse-transcribed using Taqman microRNA Reverse Transciption Kit (Applied Biosystem, Foster City, CA). Expression of mature miRNAs were examined by Real Time qPCR using TaqMan Human MicroRNA assay Kit (Applied Biosystems), and a CFX96 cycler (Biorad). MiRNA expression by each sample was evaluated in triplicate. MiRNA expression values were normalized to the expression level of a housekeeping miRNA: miR-191 [33] and reported as 2^–ΔCt^ so that it was not necessary to set a reference sample and expression levels will be comparable among laboratories.

### Transfection of miR-133b mimic and treatment with erlotinib

MiR-133b mimic and negative control oligonucleotide were obtained from QIAGEN (Hilden, Germany) and transfected into cell lines at a concentration of 20 nM with Lipofectamine RNAiMAX 0.25% (Invitrogen, Carlsbad, CA) according with the manufactures’ protocol. To assess transfection efficiency levels of miR-133b were determined by real-time PCR after 24h transfection. In addiction transfection efficiency was monitored by flow cytometry using a fluorescent negative control oligonucleotide. Erlotinib tablets (Tarceva^®^ 150 mg, UK) were pulverized and dissolved in DMSO at concentration of 10 mmol/l, and stored at -20°C. Cells were seeded in 6-well plates at 1.5 x 10^5^ cell/well in 2 ml medium. The following day cells were transfected with miR-133b mimic and negative control oligonucleotide and after 3h of transfection erlotinib was added to the cells at 2 μM. The selected dose of erlotinib reduced growth of H322 cell line which is reported as responder in the literature [34]. Cell yield, membrane EGFR expression, total expression of EGFR, ERK and pERK were evaluated after 72h of treatment. Experiments were performed in triplicate.

### Cell viability

Cells were seeded in 96-well plates at 2 x 10^4^ cells/well. The following day they were transfected with the miR-133b mimic and the negative control oligonucleotide then treated with different doses of erlotinib. After 72h treatment, WST-1 (Sigma) was added in each well (1:10 dilution). Following 4h incubation at 37°C 5% CO_2_ optical density (OD) was measured at 450 nm and 600 nm.

### Flow cytometry analysis

Cell surface expression of EGFR was evaluated by flow cytometry. After treatment cells were detached with trypsin-EDTA, pelleted then stained with 50 μl PBS containing 0.1% Live-Dead Fixable Dead Cell Stain near-IR-fluorescent reactive dye (Molecular Probes) on ice for 15 min to exclude dead cells from the analysis. After washing with PBS + 5% BSA cells were incubated on ice for 30 minutes at dark with the primary antibody anti-EGFR-Alexa Fluor 488 (528) sc-120 or isotype control antibody-Alexa Fluor 488 (Santa Cruz Biotecnology, INC.) as negative control. After washing with PBS + 5% BSA, cells were analyzed by flow cytometry with the FACSCantoII (BD) at least 10,000 events were recorded.

### Western blot analysis

Cells were lysed in lysis buffer complemented with protease-inhibitors (Cell Lysis Buffer, Cell Signaling) and incubated on ice for 40 minutes. Protein concentration was measured using the DC Protein Assay (Bio-Rad), and 50 μg of protein was separated using a 10% polyacrylamide gel and electroblotted onto nitrocellulose membranes. Membranes were immunoblotted overnight at 4°C with the following primary antibodies: rabbit anti-human EGFR (1:1000, R&D System), mouse anti-human pERK (1:1000, Cell Signalling), rabbit anti-human ERK (1:1000, Cell Signalling) and rabbit anti-human GAPDH (1:1000, Santa Cruz Biotechnologies) diluted in PBS + 2% BSA + 0.1% Tween 20. GAPDH was used as an internal control. HRP-conjugated secondary antibodies (Santa Cruz Biotechnologies) were incubated for 1 h at room temperature. Signals were detected with ECL detection reagent (Amersham) and ChemiDoc instrument (Bio-Rad).

### Statistical analysis

In absence of a-priori hypothesis, given the exploratory nature of the study, no formal sample size calculation was performed. We analysed data regarding 32 consecutive patients treated by our institution from January 2009 to June 2014. Main statistical analysis entailed a graphical exploration with boxplots to describe markers’ distribution by response groups, at first. Each graph was accompanied by a Mann-Whitney two-sided test to evaluate evidence of positional shift between marker distributions across response groups. Secondly, response prediction accuracy of each marker in turn was evaluated by means of receiver operator characteristics (ROC) curve plotting and the area under the curve (AUC) (with confidence interval) estimation; a dichotomizing cut-off was further determined by Youden’s index maximization. Kaplan-Meier survival curves were estimated to obtain median OS and PFS and compared with the log-rank test. Fisher exact test was used to determine the association between miR-133b levels and clinical parameters. Nonlinear regression, log (inhibitor) *vs*. normalized response, variable slope was used to determine IC50. Confidence intervals were calculated considering a 0.95 confidence level. *P*-values less than 0.05 were considered statistically significant. Statistical analysis was carried out using R 3.2.3 (R Foundation for Statistical Computing, Vienna, Austria) and Prism 6.0 software.

## Results

### Patients cohort

Thirty-two consecutive patients with advanced lung adenocarcinomas treated in the Oncology Unit, S. Maria Hospital, Reggio Emilia from January 2009 to June 2014 were included in the study. The median age was 64 years (range: 47–81 years). The female patients were 19 (59.4%), and male were 13 (40.6%). Patients received a median of two chemotherapy regimens (range: 1–2 regimens) before a second- or third-line treatment with erlotinib. The median overall survival (OS) of the patient cohort was 2.93 years (confidence interval, CI: 2.30–5.17 years) and median progression free survival (PFS) was 0.29 years (CI: 0.26–0.36 years). Mutations of EGFR were successfully evaluated in all but one patients: 26 patients (81.2%) exhibit wild type EGFR whereas 5 patients (15.7%) exhibit mutated EGFR (delE764-A750, L858R, L861Q). The patients with disease stabilization on erlotinib for at least 6 months were considered responders. According to this classification, 8 patients (25%) were responders, and 24 patients (75%) were non-responders to erlotinib in the analysed cohort. The PSF of responders was longer than in non-responders patients (median PFS was 1.10 years vs. 0.26 years, p<0.001). Demographical and clinical characteristics of the two groups of patients at diagnosis are reported in Table 1.

**Table 1:** Clinicopathological characteristics of NSCLC patients at diagnosis classified as responders and non-responders to erlotinib.

Characteristics		No. of patients = 32	
		Responders	Non-responders
		n = 8	n = 24
**Age at diagnosis**	**< 65 years**	2 (25%)	15 (62.5%)
	**≥ 65 years**	6 (75%)	9 (37.5%)
**Gender**	**Female**	7 (87.5%)	12 (50%)
	**Male**	1 (12.5%)	12 (50%)
**Stage**	**I-II**	0 (0%)	6 (25%)
	**III-IV**	8 (100%)	17 (70.8%)
	**NA**	0 (0%)	1 (4.2%)
**Erlotinib (TKIs)**	**2**^**nd**^ **line**	4 (50%)	18 (75%)
	**3**^**rd**^ **line**	4 (50%)	6 (25%)
**EGFR gene status**	**Wild-type**	5 (62.5%)	21 (87.5%)
	**Mutant**	2 (25%)	3 (12.5%)
	**NA**	1 (12.5%)	0 (0%)
**OS (CI)**		4.34 (3.40-nd)	2.34 (1.86–5.65)
PFS (CI)*		1.10 (0.84-nd)	0.26 (0.23–0.33)

Median OS (Overall Survival) and PFS (Progression Free Survival) obtained with Kaplan-Maier survival curve analysis are shown with CI (Confidence Interval) in years.

Nd (not determined) indicates that the survival curve confidence interval upper limit did not reached the 0.5 value during the follow up considered.

*p<0.001 by log-rank test. Fisher’s exact Test was not statistically significant for all the characteristics.

### MiRNAs expression in lung cancers specimens at diagnosis in relation to response to erlotinib

**Fig 1:**
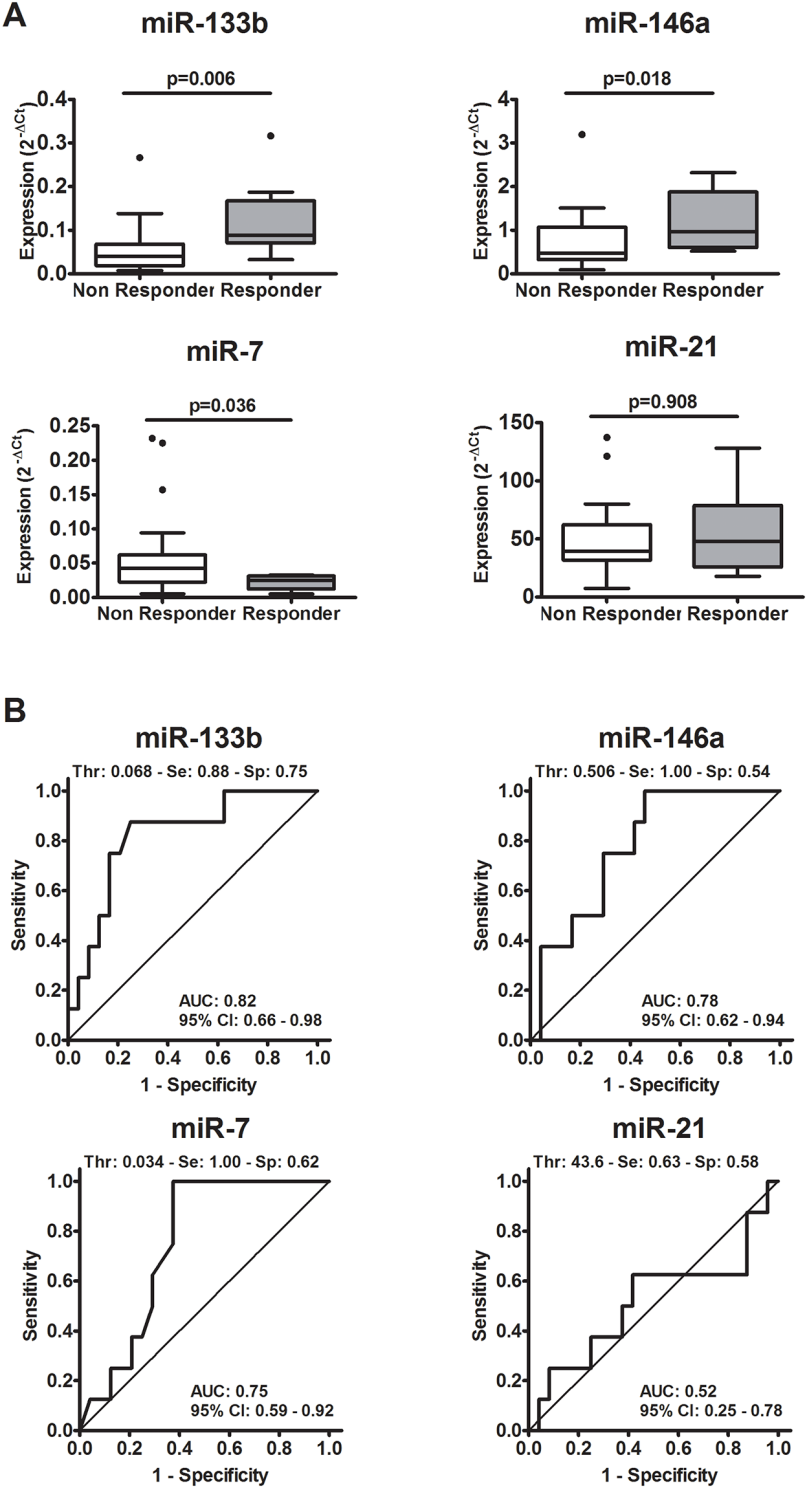
MiRNAs expression in relation to response to erlotinib. **A** Analysis of miRNA expression was investigated using TaqMan real-time quantitative PCR in NSCLC specimen from "responders" (n = 8) and "non-responders" patients (n = 24). Results are shown as normalized expression: 2^−ΔCt^ compared with Mann-Whitney test. **B** ROC curve analysis of miRNA levels to predict response to erlotinib. AUC = Area Under the Curve; CI = Confidence Interval; Thr = Threshold; Se = Sensitivity; Sp = Specificity.

To determine whether miRNAs correlated with response to erlotinib in patients with NSCLC, expression levels of EGFR-related miRNAs were analysed by real-time PCR in FFPE lung tissue samples obtained from the routine biopsies or therapeutic surgery. Four miRNAs, involved in the regulation of EGFR expression and with the highest sum in miRTarBase in 2013, were selected: miR-7, -21, -133b and -146. Expression levels of miR-7 in responders were significantly lower than in non-responder patients (*p* = 0.037), while expression levels of miR-21 were similar between the two cohorts (*p* = 0.915). In contrast, expression levels of miR-133b and miR-146a were significantly higher in responders than in non-responder patients (respectively, *p* = 0.006 and *p* = 0.018) (Fig 1A).

ROC curve analysis allowed to define accuracy of the miRNAs in predicting response to erlotinib. MiR-133b, miR-146a and miR-7 showed a diagnostic value in discriminating the two cohorts of patients (in contrast to miR-21) (Fig 1B), with the first seeming to perform a little better than the other. In particular a miR-133b level higher than 0.068 identified patients who effectively responded to erlotinib with 75% specificity and 88% sensitivity. Overall, miR-133b showed the highest differential expression between responders and non-responders (2.2 fold) and reached the lowest *p* values among the investigated miRNAs after Mann-Whitney and ROC curve analysis thus it was selected for further investigations.

The stratification of patients based on miR-133b expression level in NSCLC specimens (>0.068 n = 12; ≤0.068 n = 20) revealed that patients with higher expression of miR-133b had a longer median PFS by Kaplan-Meier curve analysis (0.48 years versus 0.27 years, *p* = 0.005).

### MiR-133b effects on sensitivity to erlotinib

To investigate whether miR-133b might modify the sensitivity to erlotinib, we analyzed the effects of the transfection of miR-133 mimic in NSCLC cell lines combined to erlotinib treatment. Two cell lines with wild-type EGFR (A549 and H1299) and resistant to erlotinib were chosen to mimic the cohorts of patients included in the study (>80% of patients had wild-type EGFR). H1299 and A549 expressed low levels of miR-133b, A549 about two fold more than H1299. The threshold cycles for miR-133b of H1299 and A549 were 36 and 35 respectively.

After 24h transfection with miR-133b mimic H1299 reached a 10^6^ fold increase and A549 reached a 10^5^ fold increase in miR-133b expression. In addition, kinetics of transfection using a fluorescent negative control oligonucleotide and flow cytometry showed that 100% cells were still transfected up to 72h. Their median fluorescence intensity after transfection declined from 24h to 72h of treatment paralleling growth rate (data not shown).

Increasing the expression of miR-133b did not affect the sensitivity to erlotinib in H1299 and A549 cell lines. The concentration of erlotinib which reduced cell viability by half (IC50) was 107 μM in negative control transfected H1299 cells (CI 86–133 μM) versus 104 μM in miR-133b mimic transfected H1299 cells (CI 83–131 μM); 79 μM in negative control transfected A549 cells (CI 68–93 μM) versus 71 μM in miR-133b mimic transfected A549 cells (CI 60–85 μM).

**Fig 2:**
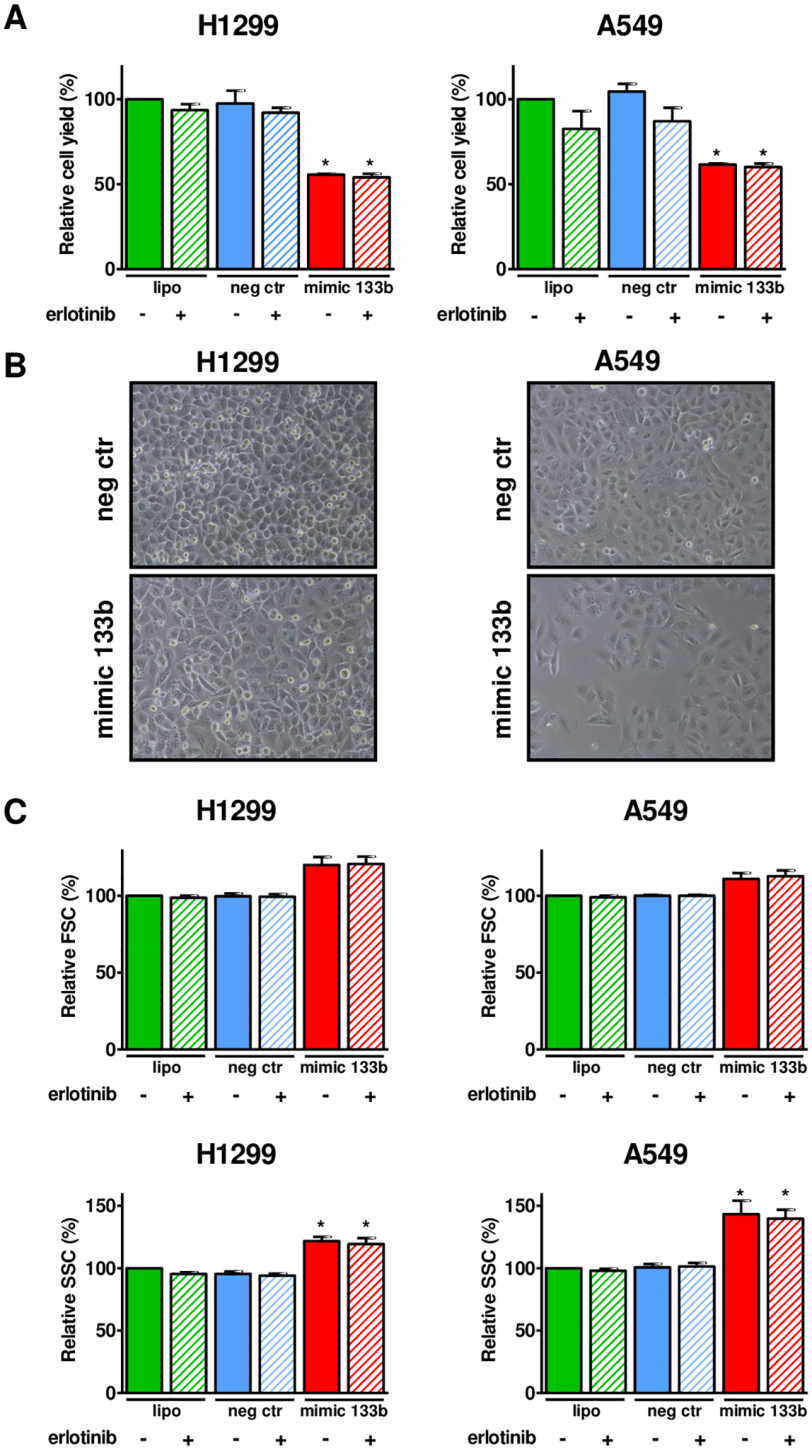
Effects of miR-133b in combination to erlotinib on NSCLC cell lines. Cells were seeded at 1.5 x 10^5^ cells/well and cultured up to 72 hours. Data are presented as mean ± SEM of three independent experiments. **A** Cell growth relative to lipofectamine-treated cells. **B** Photographs of A549 and H1299 after 72 h treatment. Magnification 400X. **C** Cell shape measured as forward scatter (FSC) shown on the top and side scatter (SSC) shown on the bottom. Note that the axes in each diagram are displayed in relative percent scale. *p<0.05 by the one sample *t* test with hypothetical value = 100.

For subsequent experiments we chose to use a clinically relevant dose of erlotinib (2 μM), comparable to that found in plasma of patients after erlotinib administration [34]. Transfection of miR-133b mimic significantly decreased cell growth in both cell lines: the growth of H1299 cells was decreased by 50% and the growth of A549 cells was decreased by 40% compared to negative control oligonucleotide- and lipofectamine-treated cells (Fig 2A). No effects were observed when cells were treated with erlotinib and negative control oligonucleotide. Moreover, the combined treatment of miR-133b mimic and erlotinib decreased cell growth equally to miR-133b mimic alone in both cell lines. Notably, effects on cell growth were detected only after 72h of treatment.

H1299 and A549 cells transfected with miR-133b mimic for 72h showed a different morphology compared to negative control oligonucleotide-transfected cells (Fig 2B) which was confirmed by changes in forward scatter and side scatter evaluated with flow cytometry (Fig 2C). Forward scatter is a measure of cell dimension. Side scatter is a measure of cell complexity. Cells treated with the combination of miR-133b mimic and erlotinib were similar to those treated with the miR-133 mimic alone.

**Fig 3:**
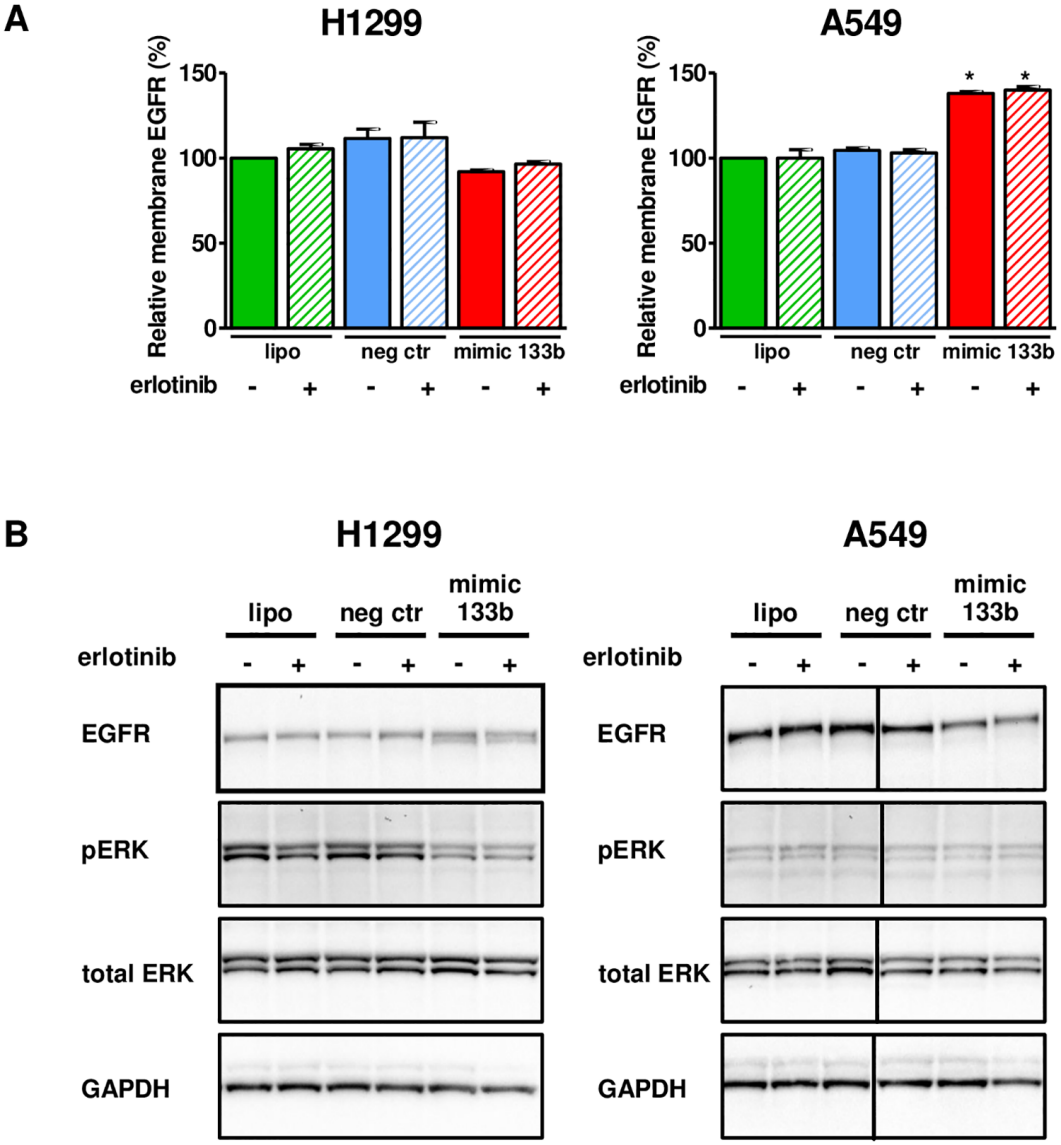
Effects of mir-133b and erlotinib on EGFR and EGFR pathway. **A** Flow cytometry to evaluate EGFR surface expression. Expression relative to lipofectamine treated cells is shown (mean ± SEM of three independent experiments is shown). *p<0.05 by the one sample *t* test with hypothetical value = 100. **B** Western blot assay to detect total EGFR, total ERK, GAPDH and phosphorylated ERK. Cropped blots are displayed. Full length blots are showed in S1 Fig. Exposure time: EGFR 200 sec, GAPDH 90 sec, pERK 420 sec, total EGFR 90 sec.

To gain insight into molecular mechanisms, we then investigated the effects of miR-133b mimic, erlotinib and their combination on the surface expression of EGFR, total expression of EGFR and EGFR downstream signaling pathway. EGFR surface expression was unexpectedly increased in A549 cell line after 72h transfection with miR-133b mimic while it did not change in H1299 cell line (Fig 3A). On the other hand, total EGFR expression resulted decreased in A549 cell line after 72h transfection with miR-133b mimic while increased in H1299 cell line. No changes were found in the levels of total ERK and phosphorylated ERK in A549 cell line, whereas ERK phosphorylation was found to be decreased in H1299 cell lines (Fig 3B). Treatment with negative control oligonucleotide did not modify total and membrane EGFR expression as well as total level of ERK and phosphorylated ERK. Treatment with erlotinib did not modify total and membrane EGFR and total ERK expression while slightly decreased phosphorylated ERK only in H1299 cell line. We did not detect differences between the effects of treatment with miR-133b mimic alone and the combined treatment of miR-133b mimic and erlotinib in both cell lines.

## Discussion

In this study we found that increased expression levels of miR-133b and miR-146a and reduced expression levels of miR-7 in lung cancer specimens were associated with higher efficacy of erlotinib as second and third line therapy in patients with NSCLC. In particular miR-133b slightly outperformed the others, suggesting that miR-133b might be a potential predictive marker of response to erlotinib treatment. In addition, increasing miR-133b expression in NSCLC cell lines led to a decreased growth but did not modify the sensitivity to erlotinib.

Our data and literature data support a tumor suppressor role for miR-133b in NSCLC. MiR-133b expression levels in NSCLC tissue has been found significantly lower compared to non-neoplastic tissue [25, 35–37]. In addition, miR-133b has been documented to decrease NSCLC cell growth, survival, migration and invasion [25, 35, 37–39]. Besides, high expression of miR-133b in NSCLC is emerging as a good prognostic factor. Patients in our cohort with higher expression of miR-133b (>0.068) showed a better PFS, whereas no differences were found in OS between patients with high and low miR-133b expression. A recent study by Chen et al revealed that high expression of miR-133b in NSCLC correlated with lower tumor stage, absence of lymph node metastasis and longer OS time of patients [36]. The work by Liu et al also showed a negative correlation between miR-133b expression levels in NCSLC and lymph node metastasis but no correlation was found with OS time of patients [25].

Since higher levels of miR-133b in NSCLC specimens were associated with longer PFS of patients under erlotinib treatment, we hypothesized that miR-133b might enhance the sensitivity to erlotinib. Instead we found that increasing the expression of miR-133b by transfecting a miR-133b mimic did not affect the sensitivity to erlotinib in A549 and H1299 cell lines. Other authors have found that increasing the expression of miR-133b caused a significant increase in the sensitivity to the EGFR TKI gefitinib in H1650 and H1975 cell lines but, in agreement with our data, not in A549 cell line [25]. We might speculate that miR-133b effects in combination with EGFR-TKIs vary according to the EGFR status of the cells: wild type (A549 and H1299) *versus* mutated (H1650 and H1975), increasing EGFR-TKIs efficacy only in case of mutated EGFR. In addition, miR-133b might be also expressed by cells of the microenvironment in the tumor specimens leading to results in vivo which cannot be fully evaluated through in vitro experiments performed on cancer cell lines.

The study of the effects of miR-133b mimic transfection on EGFR pathway revealed differences between A549 and H1299 cell lines despite a common inhibition of cell growth. This is the first study which investigated plasma membrane expression of EGFR following miR-133b mimic transfection. In H1299 cells, treatment with miR-133b alone or in combination with erlotinib did not modify the total amount of EGFR and its surface expression but decreased pERK. In A549 cells, such treatments decreased the total amount of EGFR with a parallel increase in surface EGFR, suggesting a preferential localization of EGFR on the plasma membrane probably as a resistance mechanism to miR-133b activity. In addition, in A549 cells we did not find any modification in pERK suggesting that alterations in others downstream intracellular pathways caused the inhibition of cell growth.

Interestingly, we found that enhancing miR-133b expression had inhibitory effects on cell growth only after 72h treatment, suggesting that targets of miR-133b with long half-life or activated when cells become confluent might be involved. MiR-133b can target other plasma membrane receptors in addition to EGFR based on miRTarBase: IGF1R (insulin-like growth factor 1 receptor), MET (hepatocyte growth factor receptor), CXCR4 (chemokine C-X-C receptor 4) and FGFR1 (fibroblast growth factor receptor 1). These receptors can regulate NSCLC cell growth, migration, invasiveness, metastatic potential and acquired resistance to therapies. Therefore, administration of a miR-133b mimic could impact on several key pathways involved in NSCLC supporting its potential application for NSCLC therapy.

The miR-133 family contains miR-133a and miR-133b. As shown by Wang et al, miR-133a also modulated invasiveness and proliferation of lung cancer cell lines and it downregulated multiple targets including EGFR [40]. In addition, higher miR-133a expression levels were associated with N0-N1, I-II clinical stage and better overall survival rates in NSCLC patients strengthening the predictive and therapeutic potential of the miR-133 family in NSCLC [40].

A limit of the present study is the small cohort of patients. A strong point is the translational potential. Only other three studies have investigated miRNA expression in relation to response to EGFR-TKIs [31, 41, 42] and only one of them was on patients with NSCLC with wild type EGFR. Specifically miR-200c overexpression has been reported to predict a better efficacy of EGFR-TKIs in NSCLC patients with wild type EGFR [42]. Moreover upregulation of miR-200c has been reported to enhance gefitinib sensitivity in A549 and H1299, but not in H1975 cell lines.

## Conclusion

Detecting miR-133b expression levels in tumors might help in the identification of NSCLC patients that are likely to benefit from second and third-line therapy with erlotinib. In addition gene therapy approaches aiming at increasing miR-133b levels in tumors might be promising to inhibit the growth of lung cancer cells. Studies in preclinical models are needed to demonstrate the therapeutic potential of miR-133b mimic and additional studies on larger cohorts of patients are required to validate the predictive value of miR-133b concerning response to erlotinib.

## Supporting information

S1 Fig Full-length chemiluminescent western blot images. Each membrane was cut in two halves according to the molecular weight protein standards loaded in the first and 10^th^ lanes (SeeBlue Plus2 Pre-Stained Standard, Novex, Life Technologies). The upper part containing proteins > 80 kDa was stained with rabbit anti-human EGFR antibody. The lower part containing proteins between 14 and 80 kDa was stained successively with mouse anti-human pERK, rabbit anti-human ERK and rabbit anti-human GAPDH antibodies. We made a loading mistake in A549 cells: the lysate obtained from cells transfected with the miR-133b mimic + erlotinib was loaded near the lysate obtained from cells transfected with the negative control oligonucleotide. In the manuscript the lanes were cropped and flipped to have the negative control oligonucleotide + erlotinib near the negative control oligonucleotide.
(TIF)

## References

[1] Travis WD, Brembilla E, Nicholson AG, Yatabe Y, Austin JH, Beasley MB, et al The 2015 World Health Organization Classification of Lung Tumors: Impact of Genetic, Clinical and Radiologic Advances Since the 2004 Classification. J Thorac Oncol. 2015;10(9):1243–60. doi: 10.1097/JTO.0000000000000630 26291008 10.1097/JTO.0000000000000630

[2] Sharma SV, Bell D, Settleman J, Haber DA. Epidermal growth factor receptor mutations in lung cancer. Nat Rev Cancer. 2007;7(3):169–81. doi: 10.1038/nrc2088 17318210 10.1038/nrc2088

[3] Sequist LV, Joshi V, Jänne PA, Muzikansky A, Fidias P, Meyerson M, et al Response to treatment and survival of patients with non-small cell lung cancer undergoing somatic EGFR mutation testing. Oncologist. 2007;12(1):90–8. 17285735 10.1634/theoncologist.12-1-90

[4] Korpanty GJ, Graham D, Vincent MD, Leighl NB. Biomarkers That Currently Affect Clinical Practice in Lung Cancer: EGFR, ALK, MET, ROS-1, and KRAS. Front Oncol. 2014;11(4):204. 10.3389/fonc.2014.00204 PMC4127527 25157335

[5] Muhsin M, Graham J, Kirkpatrick P. Gefitinib. Nat Rev Drug Discov. 2003;2(7):515–6. doi: 10.1038/nrd1136 12841190 10.1038/nrd1136

[6] Dowell J, Minna J, Kirkpatrick P. Erlotinib hydrochloride. Nat Rev Drug Discov. 2005;4(1):13–4. doi: 10.1038/nrd1612 15690599 10.1038/nrd1612

[7] Hirsh V. Afatinib (BIBW 2992) development in non-small-cell lung cancer. Future Oncol. 2011;7(7):817–25. doi: 10.2217/fon.11.62 21732753 10.2217/fon.11.62

[8] Fukuoka M, Wu Y, Thongprasert S, Sunpaweravong P, Leong SS, Sriuranpong V, et al Biomarker analyses and final overall survival results from a phase III, randomized, open-label, first-line study of gefitinib versus carboplatin/paclitaxel in clinically selected patients with advanced non-small-cell lung cancer in Asia (IPASS). J Clin Oncol 2011;29(21):2866–74. Epub 2011 Jun 13. doi: 10.1200/JCO.2010.33.4235 21670455 10.1200/JCO.2010.33.4235

[9] Ciuleanu T, Stelmakh L, Cicenas S, Miliauskas S, Grigorescu AC, Hillenbach C, et al Efficacy and safety of erlotinib versus chemotherapy in second-line treatment of patients with advanced, non-small-cell lung cancer with poor prognosis (TITAN): a randomised multicentre, open-label, phase 3 study. Lancet Oncol 2012;13(3):300–8. Epub 2012 Jan 24. doi: 10.1016/S1470-2045(11)70385-0 22277837 10.1016/S1470-2045(11)70385-0

[10] Dziadziuszko R, Holm F, Varella-Garcia M, Bunn PA Jr. Selecting lung cancer patients for treatment with epidermal growth factor receptor tyrosine kinase inhibitors by immunohistochemistry and fluorescence in situ hybridization—why, when, and how? Clin Cancer Res 2006;12(14):4409–15. doi: 10.1158/1078-0432.CCR-06-0087 16857819 10.1158/1078-0432.CCR-06-0087

[11] Dziadziuszko R, Holm B, Skov BG, Osterlind K, Sellers MV, Franklin WA, et al Epidermal growth factor receptor gene copy number and protein level are not associated with outcome of non-small-cell lung cancer patients treated with chemotherapy. Ann Oncol 2007;18(3):447–52. doi: 10.1093/annonc/mdl407 17082511 10.1093/annonc/mdl407

[12] Wu YL, Zhong W, Li LY, Zhang XT, Zhang L, Zhou CC, et al Epidermal growth factor receptor mutations and their correlation with gefitinib therapy in patients with non-small cell lung cancer: a meta-analysis based on updated individual patient data from six medical centers in mainland China. J Thorac Oncol 2007;2(5):430–9. doi: 10.1097/01.JTO.0000268677.87496.4c 17473659 10.1097/01.JTO.0000268677.87496.4c

[13] Zhou C, Wu Y, Chen G, Feng J, Liu XQ, Wang C, et al Erlotinib versus chemotherapy as first-line treatment for patients with advanced EGFR mutation-positive non-small-cell lung cancer (OPTIMAL, CTONG-0802): a multicentre, open-label, randomised, phase 3 study. Lancet Oncol 2011;12(8):735–42. Epub 2011 Jul 23. doi: 10.1016/S1470-2045(11)70184-X 21783417 10.1016/S1470-2045(11)70184-X

[14] Mok TS, Wu Y, Thongprasert S, Yang CH, Chu DT, Saijo N, et al Gefitinib or carboplatin-paclitaxel in pulmonary adenocarcinoma. N Engl J Med 2009;361(10):947–57. Epub 2009 Aug 19. doi: 10.1056/NEJMoa0810699 19692680 10.1056/NEJMoa0810699

[15] Maemondo M, Inoue A, Kobayashi K, Sugawara S, Oizumi S, Isobe H, et al Gefitinib or chemotherapy for non-small-cell lung cancer with mutated EGFR. N Engl J Med 2010;362(25):2380–8. doi: 10.1056/NEJMoa0909530 20573926 10.1056/NEJMoa0909530

[16] Mitsudomi T, Motita S, Yatabe Y, Negoro S, Okamoto I, Tsurutani J, et al Gefitinib versus cisplatin plus docetaxel in patients with non-small-cell lung cancer harbouring mutations of the epidermal growth factor receptor (WJTOG3405): an open label, randomised phase 3 trial. Lancet Oncol 2010;11(2):121–8. Epub 2009 Dec 18. doi: 10.1016/S1470-2045(09)70364-X 20022809 10.1016/S1470-2045(09)70364-X

[17] Garassino MC, Martelli O, Broggini M, Farina G, Veronese S, Rulli E, et al Erlotinib versus docetaxel as second-line treatment of patients with advanced non-small-cell lung cancer and wild-type EGFR tumours (TAILOR): a randomised controlled trial. Lancet Oncol 2013;14(10):981–8. Epub 2013 Jul 22. doi: 10.1016/S1470-2045(13)70310-3 23883922 10.1016/S1470-2045(13)70310-3

[18] Rosell R, Carcereny E, Gervais R, Vergnenegre A, Massuti B, Felip E, et al Erlotinib versus standard chemotherapy as first-line treatment for European patients with advanced EGFR mutation-positive non-small-cell lung cancer (EURTAC): a multicentre, open-label, randomised phase 3 trial. Lancet Oncol. 2012;13(3):239–46. Epub 2012 Jan 26. doi: 10.1016/S1470-2045(11)70393-X 22285168 10.1016/S1470-2045(11)70393-X

[19] Han JY, Park K, Kim SW, Lee DH, Kim HY, Kim HT, et al First-SIGNAL: first-line single-agent iressa versus gemcitabine and cisplatin trial in never-smokers with adenocarcinoma of the lung. J Clin Oncol 2012;30(10):1122–8. Epub 2012 Feb 27. doi: 10.1200/JCO.2011.36.8456 22370314 10.1200/JCO.2011.36.8456

[20] Bronte G, Terrasi M, Rizzo S, Sivestris N, Ficorella C, Cajozzo M, et al EGFR genomic alterations in cancer: prognostic and predictive values. Front Biosci (Elite Ed). 2011;1(3):879–87. 10.2741/e296 21622099

[21] Chen CZ. MicroRNAs as oncogenes and tumor suppressors. N Engl J Med. 2005;353(17):1768–71. doi: 10.1056/NEJMp058190 16251533 10.1056/NEJMp058190

[22] Takamizawa J, Konishi H, Yanagisawa K, Tomida S, Osada H, Endoh H, et al Reduced expression of the let-7 microRNAs in human lung cancers in association with shortened postoperative survival. Cancer Res. 2004;64(11):3753–6. doi: 10.1158/0008-5472.CAN-04-0637 15172979 10.1158/0008-5472.CAN-04-0637

[23] Weiss GJ, Bemis L, Nakajima E, Sugita M, Birks DK, Robinson WA, et al EGFR regulation by microRNA in lung cancer: correlation with clinical response and survival to gefitinib and EGFR expression in cell lines. Ann Oncol 2008;19(6):1053–9. Epub 2008 Feb 27. doi: 10.1093/annonc/mdn006 18304967 10.1093/annonc/mdn006

[24] Wang Y, Wang X, Zhang J, Sun G, Luo H, Kang C, et al MicroRNAs involved in the EGFR/PTEN/AKT pathway in gliomas. J Neurooncol 2012;106(2):217–24. Epub 2011 Aug 13. doi: 10.1007/s11060-011-0679-1 21842313 10.1007/s11060-011-0679-1

[25] Liu L, Shao X, Gao W, Zhang Z, Liu P, Wang R, et al MicroRNA-133b inhibits the growth of non-small-cell lung cancer by targeting the epidermal growth factor receptor. FEBS J. 2012;279:3800–12. Epub 2012 Sep 11. doi: 10.1111/j.1742-4658.2012.08741.x 22883469 10.1111/j.1742-4658.2012.08741.x

[26] Yanaihara N, Caplen N, Bowman E, Seike M, Kumamoto K, Yi M, et al Unique microRNA molecular profiles in lung cancer diagnosis and prognosis. Cancer Cell 2006;9(3):189–98. doi: 10.1016/j.ccr.2006.01.025 16530703 10.1016/j.ccr.2006.01.025

[27] Raponi M, Dossey L, Jatkoe T, Wu X, Chen G, Fan H, et al MicroRNA classifiers for predicting prognosis of squamous cell lung cancer. Cancer Res 2009;69(14):5776–83. Epub 2009 Jul 7. doi: 10.1158/0008-5472.CAN-09-0587 19584273 10.1158/0008-5472.CAN-09-0587

[28] Seike M, Goto A, Okano T, Bowman ED, Schetter AJ, Horikawa I, et al MiR-21 is an EGFR-regulated anti-apoptotic factor in lung cancer in never-smokers. PNAS. 2009;106(29):12085–90. Epub 2009 Jul 13. doi: 10.1073/pnas.0905234106 19597153 10.1073/pnas.0905234106 PMC2715493

[29] Rabinowits G, Gerçel-Taylor C, Day JM, Taylor DD, Kloecker GH. Exosomal microRNA: a diagnostic marker for lung cancer. Clin Lung Cancer. 2009;10(1):42–6. doi: 10.3816/CLC.2009.n.006 19289371 10.3816/CLC.2009.n.006

[30] Landi MT, Zhao Y, Rotunno M, Koshiol J, Liu H, Bergen AW, et al MicroRNA expression differentiates histology and predicts survival of lung cancer. Clin Cancer Res. 2010;16(2):430–41. Epub 2010 Jan 12. doi: 10.1158/1078-0432.CCR-09-1736 20068076 10.1158/1078-0432.CCR-09-1736 PMC3163170

[31] Yan G, Yao R, Tang D, Qiu T, Shen Y, Jiao W, et al Prognostic significance of microRNA expression in completely resected lung adenocarcinoma and the associated response to erlotinib. Med Oncol 2014;31(10):203 doi: 10.1007/s12032-014-0203-5 25192889 10.1007/s12032-014-0203-5

[32] Edge S, Byrd D, Compton CC, Fritz AG, Greene FL, Trotti A. AJCC Cancer Staging Manual 7th Edition Edition Chicago: Springer; 2010.

[33] Croci S, Zerbini A, Boiardi L, Muratore F, Bisagni A, Nicoli D, et al MicroRNA markers of inflammation and remodelling in temporal arteries from patients with giant cell arteritis. Ann Rheum Dis. 2016;75(8):1527–33. Epub 2015 Sep 4. doi: 10.1136/annrheumdis-2015-207846 26342092 10.1136/annrheumdis-2015-207846

[34] Li T, Ling Y, Goldman ID, Perez-Soler R. Schedule-Dependent Cytotoxic Synergism of Pemetrexed and Erlotinib in Human Non–Small Cell Lung Cancer Cells. Clinical Cancer Research. 2007;13(11):3413–22. doi: 10.1158/1078-0432.CCR-06-2923 17545550 10.1158/1078-0432.CCR-06-2923

[35] Zhang YQ, Wang W, Xue JX, Xu Y, Fan P, Caughey BA, et al MicroRNA Expression Profile on Solid Subtype of Invasive Lung Adenocarcinoma Reveals a Panel of Four miRNAs to Be Associated with Poor Prognosis in Chinese Patients. J Cancer. 2016;7(12):1610–20. doi: 10.7150/jca.14923 27698898 10.7150/jca.14923 PMC5039382

[36] Chen SW, Wang T, Tian YH, Zheng YG. Down-regulation of microRNA-126 and microRNA-133b acts as novel predictor biomarkers in progression and metastasis of non small cell lung cancer. Int J Clin Exp Pathol. 2015;8(11):14983–8. 26823832 PMC4713618

[37] Crawford M, Batte K, Yu L, Wu X, Nuovo GJ, Marsh CB, et al MicroRNA 133B targets pro-survival molecules MCL-1 and BCL2L2 in lung cancer. Biochem Biophys Res Commun 2009;388(3):483–189. Epub 2009 Aug 3. doi: 10.1016/j.bbrc.2009.07.143 19654003 10.1016/j.bbrc.2009.07.143 PMC2824514

[38] Zhen Y, Liu J, Huang Y, Wang Y, Li W, Wu J. MiR-133b inhibits cell growth, migration, and invasion by targeting MMP9 in non-small cell lung cancer. Oncol Res 2016 doi: 10.3727/096504016X14800889609439 27938481 10.3727/096504016X14800889609439 PMC7840966

[39] Yang X, Lei P, Huang Y, Zhang Z, Zhang Y. MicroRNA-133b inhibits the migration and invasion of non small cell lung cancer cells via targeting FSCN1. Oncol Lett 2016;12(3):3619–25. doi: 10.3892/ol.2016.5044 27900045 10.3892/ol.2016.5044 PMC5104139

[40] Wang Y, Li J, Chen H, Mo Y, Ye H, Luo Y, et al Down-regulation of miR-133a as a poor prognosticator in non-small cell lung cancer. Gene 2016;591(2):333–7. Epub 2016 Jun 6. doi: 10.1016/j.gene.2016.06.001 27282282 10.1016/j.gene.2016.06.001

[41] Bryant JL, Britson J, Balko JM, Willian M, Timmons R, Frolov A, et al A microRNA gene expression signature predicts response to erlotinib in epithelial cancer cell lines and targets EMT. Br J Cancer 2012;106(1):148–56. Epub 2011 Nov 1. doi: 10.1038/bjc.2011.465 22045191 10.1038/bjc.2011.465 PMC3251842

[42] Li J, Li X, Ren S, Chen X, Zhang Y, Zhou F, et al miR-200c overexpression is associated with better efficacy of EGFR-TKIs in non-small cell lung cancer patients with EGFR wild-type. Oncotarget. 2014;5(17):7902–16. doi: 10.18632/oncotarget.2302 25277203 10.18632/oncotarget.2302 PMC4202169

