# Supplementary figures and images for: Higher expression of miR-133b is associated with better efficacy of erlotinib as the second or third line in non-small cell lung cancer patients
Source: PLoS One. 2018 Apr 24;13(4):e0196350. doi: 10.1371/journal.pone.0196350 (PMC5916492; doi:10.1371/journal.pone.0196350)

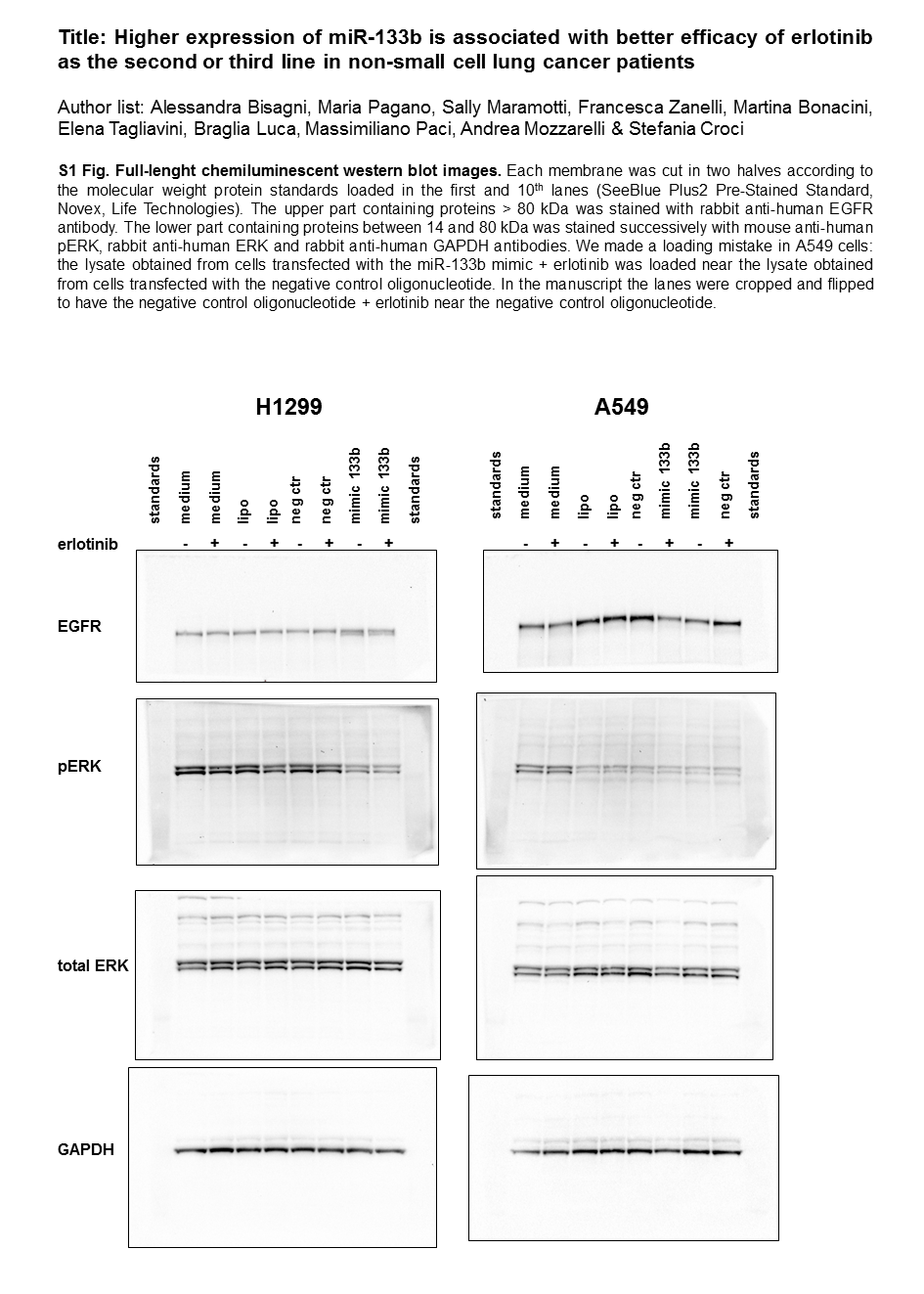

Supplement: S1 Fig — Each membrane was cut in two halves according to the molecular weight protein standards loaded in the first and 10th lanes (SeeBlue Plus2 Pre-Stained Standard, Novex, Life Technologies). The upper part containing proteins > 80 kDa was stained with rabbit anti-human EGFR antibody. The lower part containing proteins between 14 and 80 kDa was stained successively with mouse anti-human pERK, rabbit anti-human ERK and rabbit anti-human GAPDH antibodies. We made a loading mistake in A549 cells: the lysate obtained from cells transfected with the miR-133b mimic + erlotinib was loaded near the lysate obtained from cells transfected with the negative control oligonucleotide. In the manuscript the lanes were cropped and flipped to have the negative control oligonucleotide + erlotinib near the negative control oligonucleotide. (TIF) [file pone.0196350.s001.TIF]
